# The Contribution of Efflux Systems to Levofloxacin Resistance in *Stenotrophomonas maltophilia* Clinical Strains Isolated in Warsaw, Poland

**DOI:** 10.3390/biology11071044

**Published:** 2022-07-12

**Authors:** Olga M. Zając, Stefan Tyski, Agnieszka E. Laudy

**Affiliations:** 1 Department of Pharmaceutical Microbiology, Medical University of Warsaw, 02-097 Warsaw, Poland; olga.zajac@wum.edu.pl (O.M.Z.); s.tyski@nil.gov.pl (S.T.); 2 Department of Antibiotics and Microbiology, National Medicines Institute, 00-725 Warsaw, Poland

**Keywords:** antibiotic susceptibility, CCCP, efflux pumps, efflux pump inhibitors, efflux system regulatory genes, EPI, levofloxacin resistance, non-fermentative rods, PAβN, reserpine

## Abstract

**Simple Summary:**

Fluoroquinolones, mainly levofloxacin, are considered an alternative treatment option of *Stenotrophomonas maltophilia* infections to trimethoprim/sulfamethoxazole. However, an increase in the number of levofloxacin-resistant strains is observed worldwide. The fluoroquinolone resistance in *S. maltophilia* is usually caused by an overproduction of various multidrug efflux pumps, which are able to extrude antibiotics and chemotherapeutics from the bacterial cells. The purpose of the study was to analyze the contribution of efflux systems to levofloxacin resistance in *S. maltophilia* clinical strains, isolated in Warsaw, by phenotypic and molecular methods. Previously, the occurrence of genes encoding various ten efflux pumps was shown in 94 studied isolates. Additionally, 44 of 94 isolates demonstrated reduction in susceptibility to levofloxacin. In this study, in the presence of efflux pump inhibitors, an increase in levofloxacin susceptibility was observed in 13 isolates. The overexpression of genes encoding two efflux pump system, such as SmeDEF and Sme VWX (in five and one isolate, respectively), was demonstrated. Sequencing analysis revealed an amino acid change in the local regulators of these efflux pump operons. Our data indicate that the overproduction of the SmeVWX efflux system, unlike SmeDEF, plays a significant role in the levofloxacin resistance of the clinical isolates.

**Abstract:**

Levofloxacin is considered an alternative treatment option of *Stenotrophomonas maltophilia* infections to trimethoprim/sulfamethoxazole. The fluoroquinolone resistance in *S. maltophilia* is usually caused by an overproduction of efflux pumps. In this study, the contribution of efflux systems to levofloxacin resistance in *S. maltophilia* clinical isolates was demonstrated using phenotypic (minimal inhibitory concentrations, MICs, of antibiotics determination ± efflux pump inhibitors, EPIs) and molecular (real-time polymerase-chain-reaction and sequencing) methods. Previously, the occurrence of genes encoding ten efflux pumps was shown in 94 studied isolates. Additionally, 44/94 isolates demonstrated reduction in susceptibility to levofloxacin. Only 5 of 13 isolates (with ≥4-fold reduction in levofloxacin MIC) in the presence of EPIs showed an increased susceptibility to levofloxacin and other antibiotics. The overexpression of *smeD* and *smeV* genes (in five and one isolate, respectively) of 5 tested efflux pump operons was demonstrated. Sequencing analysis revealed 20–35 nucleotide mutations in local regulatory genes such as *smeT* and *smeRv*. However, mutations leading to an amino acid change were shown only in *smeT* (Arg123Lys, Asp182Glu, Asp204Glu) for one isolate and in *smeRv* (Gly266Ser) for the other isolate. Our data indicate that the overproduction of the SmeVWX efflux system, unlike SmeDEF, plays a significant role in the levofloxacin resistance.

## 1. Introduction

*Stenotrophomonas maltophilia* is an opportunistic pathogen that is especially dangerous for patients with cystic fibrosis, immunodeficiencies, cancer (particularly lung cancer), and those exposed to immunosuppressive therapy, mechanical ventilation, and catheters. The critically ill patients from intensive care units, as well as patients hospitalized for long periods, especially after broad-spectrum antibiotic therapy, are most exposed to *S. maltophilia* infections [1,2,3]. It is worth emphasizing that infections caused by *S. maltophilia* are characterized by a high mortality rate, even up to 69% in patients with bacteremia. Infections occur in both adults and children. Although it is mainly a nosocomial pathogen, community-acquired infections are also observed. An increase in the incidence of *S. maltophilia* infections in the global population, from 1.3% to 1.7%, was observed between 2007–2012 [4].

The most important problem in the treatment of infections caused by *S. maltophilia* is an intrinsic resistance of this bacterium to a wide range of antibiotics and chemotherapeutic agents. The drug of choice for the treatment of *S. maltophilia* infections is trimethoprim/sulfamethoxazole [2,5]. However, a slow but continuous increase in the number of the trimethoprim/sulfamethoxazole-resistant strains is observed worldwide. Resistance rates in the world usually range between 1–20%; however, higher resistance levels have been reported both in Europe (Spain: 27%) and in other continents (Turkey: 10–15%, Taiwan: up to 25%, China: 30–48%) [6,7,8]. Other therapeutic options include the use of fluoroquinolones, minocycline, and ticarcillin/clavulanate. Recently, it has been revealed that fluoroquinolones, an alternative treatment, are equally as effective as trimethoprim/sulfamethoxazole [9,10,11]. Levofloxacin was most frequently used among fluoroquinolones (187 of 331, 56.5%), followed by ciprofloxacin (114 of 331, 34.4%) [10]. Generally, resistance rates to levofloxacin of *S. maltophilia* strains are relatively similar or higher than that of trimethoprim/sulfamethoxazole and range between 3% and 30% [9,12,13]. In our previous publication, all 94 investigated *S. maltophilia* clinical isolates were susceptible to trimethoprim/sulfamethoxazole and minocycline, but 7 of them were resistant and 37 were intermediately susceptible to levofloxacin [14].

The resistance to fluoroquinolones in *S. maltophilia* strains is caused by two main mechanisms: an overexpression of multidrug-resistant (MDR) efflux pumps and chromosomally encoded Qnr proteins (SmQnr). Five families (superfamilies) of efflux pumps are described. One of them, ABC (ATP-binding cassette) family, gets its energy from ATP disruption. The other 4 families are proton pumps. Of these, the MATE (multidrug and toxic compound extrusion) family may also take advantage of the differences in sodium ion concentration gradients. It is worth emphasizing that the pumps from the RND (resistance-nodulation-division) family are of the greatest importance for the resistance of Gram-negative bacteria. Unlike the others, pumps from the RND family can extrude antimicrobial agents from various chemical groups from the cells. Twelve MDR efflux systems have been identified in *S. maltophilia* so far. These systems were classified into three families of MDR efflux pumps: RND, ABC, and MFS (major facilitator superfamily). The following efflux systems, SmeDEF [15], SmeMN [14], SmeABC [16], SmeYZ [17], SmeIJK [17], SmeVWX [18], SmeOP [19], and SmeGH [20], are described from the RND family. The other four efflux pumps, SmrA [21], MacABCsm [22], SmaCDEF, and SmaAB [23], belong to the ABC family. Moreover, in *S. maltophilia*, the presence of the EmrCABsm system [24] from the MFS family has been described and the FuaABC pump [25] is unclassified as of yet. Fluoroquinolones, including levofloxacin, are substrates for most of these RND efflux systems. The following fluoroquinolone-removing efflux pumps, 5 from the RND family—smeDEF [15], SmeABC [16], SmeVWX [18], SmeGH [20], SmeIJK [17], and one from the ABC family—SmrA [21,26], have been identified so far. The best investigated efflux systems of *S. maltophilia* are SmeDEF and SmeVWX. Both of these systems are responsible for antibiotics extruding, except fluoroquinolones, as follows: SmeDEF—trimethoprim/sulfamethoxazole, chloramphenicol, macrolides, tetracycline, and tigecycline, SmeVWX—trimethoprim/sulfamethoxazole, and chloramphenicol. The RND efflux systems that occur in *S. maltophilia*, as in other Gram-negative rods, are encoded by genes organized in operons that are located in bacterial chromosomes. The relationship between operon overexpression of these two systems and the resistance of strains to fluoroquinolones has been demonstrated [15,18,27,28]. Expression of both of the above-mentioned efflux systems operons in *S. maltophilia* is regulated by local regulatory genes. The *smeDEF* operon is negatively regulated by the TetR-type transcriptional local repressor SmeT [29], whereas the *smeVWX* operon is regulated by the LysR-type transcription regulator-SmeRv [18]. Like many LysR-type regulators, SmeRv could act as a negative or positive regulator, depending on the presence of an activator ligand [18]. It has been described that mutations in *smeT* and *smeRv* regulatory genes are responsible for the overexpression of *smeDEF* and *smeVWX* efflux systems operons, respectively [30]. The third fluoroquinolone-removal efflux system is SmeABC, which also extrudes aminoglycosides and β-lactams [26]. A two-component regulatory system (TCS), SmeRS, is involved in expression of the *smeABC* operon [31]. The overproduction of the SmeGH efflux system leads to resistance to β-lactams, quinolones, tetracycline, and polymyxin B [20,32], while the SmeIJK efflux system confers resistance to aminoglycosides, tetracycline, and minocycline, but it also has a role in adaptation to envelope stress [33]. The local regulatory systems of SmeGH and SmeIJK have not been characterized yet. The other important fluoroquinolone-resistance mechanism in *S. maltophilia* is the presence of *qnr* genes encoding the pentapeptide repeat proteins, which protects topoisomerase IV and gyrase from fluoroquinolones binding [26,34]. The mechanism associated with Smqnr proteins usually contributes to the low level of fluoroquinolone resistance in *S. maltophilia* [34]. However, Wu et al. demonstrated the high level of *qnr* genes transcription in 39% of fluoroquinolone-resistant clinical strains of *S. maltophilia* [35]. It can be underlined that *S. maltophilia* is the only known bacterium in which resistance to quinolones is not associated with mutations in genes encoding topoisomerases [36].

In the previous article, we performed phenotypic and molecular characterization of the collection of 94 *S. maltophilia* clinical isolates, including the prevalence of efflux systems. The investigation revealed the presence of *smeD* and *smeH* genes in all isolates, *smeW* in 93 out of 94 isolates, *smeI* or *smeK* in 86, and *smeA* or *smeB* in 72 isolates [14]. Among them, 7 isolates were resistant, and 37 isolates demonstrated an intermediate level of susceptibility to levofloxacin.

The aim of this study was to investigate the role of RND efflux systems in resistance to levofloxacin in *S.maltophilia* clinical strains isolated in Warsaw, Poland. The studies were conducted by analyzing the effect of known efflux pump inhibitors (EPIs) on the decrease in minimal inhibitory concentration (MIC) values of levofloxacin and other MDR efflux systems substrates, as well as by analyzing the expression of genes encoding the efflux systems that remove fluoroquinolones. In addition, we studied the local regulatory genes of RND efflux systems by sequencing DNA templates.

## 2. Materials and Methods

### 2.1. Bacterial Strains

The investigation was performed on the collection of 94 non-duplicate *S. maltophilia* clinical isolates. Isolates were derived from January 2010 to October 2013 from a variety of clinical materials obtained during routine diagnostic testing of adult patients in microbiology laboratories. All clinical materials from which the tested isolates were derived are listed in our previous publication [14]. Most of the isolates (*n* = 27) were obtained from blood samples. In our previous article, all of these isolates were characterized by determination of the susceptibility profiles for three anti-*S. maltophilia* agents (including levofloxacin) and tested for the presence of genes encoding efflux pumps from the RND and ABC families. Moreover, the genetic relationship between clinical isolates and their similarity to the strains isolated in other countries was investigated [14]. All isolates were stored in Luria Bertani broth (BioMaxima SA, Lublin, Poland) with 20% glycerol at −80 °C until analysis. *Escherichia coli* ATCC 25922, *S. maltophilia* ATCC 13637, *S. maltophilia* ATCC 12714, and *S. maltophilia* 67/2013 clinical isolate, were used as reference strains in this study.

### 2.2. Determination of the MICs of Antibiotics with and without Efflux Pump Inhibitors

The minimal inhibitory concentrations of levofloxacin and gentamicin (both from Sigma, St. Louis, MO, USA), in the presence or absence of EPIs, were estimated using a 2-fold broth microdilution method in Mueller-Hinton II (MH II) broth medium (Becton, Dickinson and Company, Franklin Lakes, NJ, USA), according to the Clinical and Laboratory Standards Institute (CLSI) guidelines [37]. The MIC values of trimethoprim/sulfamethoxazole, polymyxin B, erythromycin, tigecycline and chloramphenicol, in the presence or absence of EPIs, were estimated using E-tests (Liofilchem srl, Roseto deli Abruzzi, Italy) on Mueller-Hinton II agar medium (Becton, Dickinson and Company, Franklin Lakes, NJ, USA) according to the manufacturer’s instructions [38]. In this study, the following efflux pump inhibitors were used: cyanide 3-chlorophenylhydrazone (CCCP), phenylalanine-arginine β-naphthylamide (PAβN), and reserpine (all from Sigma, St. Louis, MO, USA). CCCP and reserpine were dissolved in dimethyl sulfoxide (DMSO), while PAβN was dissolved in water. The final concentrations of EPIs in both MH II media, broth, and agar were 25 mg/L for PAβN and reserpine and 1 mg/L for CCCP. The used concentrations of EPIs were below a quarter of the MIC values of these inhibitors as determined for the tested isolates.

The results of the susceptibility tests of the studied isolates, in the presence or absence of EPIs, were evaluated after incubation at 35 °C for 18 h. The MICs of antimicrobial agents were interpreted according to the CLSI criteria [39]. Quality control of the MICs determination was performed using the reference strain of *Escherichia coli* ATCC 25922. At least a 4-fold reduction in the MIC values of antimicrobial agent after addition PAβN, reserpine, or CCCP was considered significant [40]. Such a significant decrease in drug susceptibility in the presence of at least one of the EPIs was interpreted as the likely contribution of efflux pumps to antibiotic resistance of the studied isolate.

### 2.3. RNA Preparation and Quantitative Real-Time PCR (qPCR)

The expression levels of *smeD, smeA, smeI, smeV,* and *smeG* were assessed by real-time PCR using specific primers listed in Table 1. Triplicate cell suspensions were prepared and inoculated in Luria-Bertani (LB) broth (Becton, Dickinson and Company, Franklin Lakes, NJ, USA). After achieving a logarithmic-phase of *S. maltophilia* growth (optical density, OD_600_ = 0.6), total RNA was extracted using a RNeasy mini-kit (Qiagen, Germantown, MD, USA). DNA was removed using DNA-free kit (Invitrogen, Thermo Fisher Scientific, Waltham, MA, USA). RNA was reverse transcribed to cDNA using the SuperScript III first-strand synthesis system for RT-PCR (Invitrogen, Thermo Fisher Scientific, Waltham, MA, USA) according to manufacturer’s instructions. qPCR was carried out in triplicate on each sample using a CFX96 touch Deep Well real-time PCR system (Bio-Rad, Hercules, CA, USA) and SYBR Green JumpStart Taq Ready Mix kit (Sigma, St. Louis, MO, USA). Amplification conditions were as follows: 94 °C for 10 min, followed by 40 cycles at 94 °C for 15 s and 60 °C for 30 s. The expression levels of target genes were normalized using the *16S rDNA, rpo,* and *gyr* housekeeping genes as endogenous controls and taking primer efficiency into account. Results were obtained as a relative expression in comparison to levofloxacin-susceptible *S. maltophilia* 67/2013 clinical isolate from Warsaw’s hospital. This isolate was chosen as a reference due to its high susceptibility to levofloxacin (MIC_levofloxacin_ = 1 mg/L), the relatively low MIC value of gentamicin (MIC_gentamicin_ = 64 mg/L), and the presence of all eight known efflux systems from the RND family, taking into account pulsed field gel electrophoresis (PFGE) results [14].

### 2.4. Amplification and Sequence Analysis of Efflux System Regulatory Genes

The total DNA of selected clinical isolates was extracted using a Genomic Mini Kit (A&A Biotechnology, Gdynia, Poland). The PCR reactions were performed using Maxima Hot Start Taq DNA polymerase (Thermo Scientific, Thermo Fisher Scientific, Waltham, MA, USA) with the following amplification parameters: 95 °C for 4 min, followed by 25 cycles of 30 s at 95 °C, 30 s at 58 °C for *smeRv*, or 30 s at 59 °C for *smeT*, 75 s at 72 °C, and a final extension of 5 min at 72 °C. The sequences of the primers were designed for this project based on gene sequences available in the GenBank National Centre for Biotechnology Information (NCBI; https://www.ncbi.nlm.nih.gov/, accessed on 1 April 2021) or derived from publications. All primers are listed in Table 1. Sequencing of the obtained DNA templates was carried out in the Laboratory of DNA Sequencing and Oligonucleotide Synthesis Institute of Biochemistry and Biophysics, Polish Academy of Science in Warsaw (IBB PAS). The received DNA sequences were analyzed using Vector NTI Advance 11 software (Invitrogen, Thermo Fisher Scientific, Waltham, MA, USA) and compared with the efflux regulatory gene sequences of the reference *S. maltophilia* 67/2013 strain.

### 2.5. Whole Genome Sequencing (WGS)

Whole genome sequencing was performed in the Laboratory of DNA Sequencing and Oligonucleotide Synthesis IBB PAS in Warsaw using MiSeq system (Illumina Sequencing Technology) (Illumina, San Diego, CA, USA). The detection of sequences encoding *smeRv* regulatory genes was performed in IBB PAS using PROKKA software v1.14.6. The obtained DNA sequences were compared with the DNA sequences of the reference *S. maltophilia* 67/2013 strain using Vector NTI Advance 11 software (Invitrogen, Thermo Fisher Scientific, Waltham, MA, USA).

## 3. Results

### 3.1. Effect of EPIs on the MIC Values of Antibiotics

The results of the impact of three EPIs on the MICs of levofloxacin and gentamicin investigated for 94 clinical isolates are provided in Table S1 in Supplementary Materials. A 4-fold reduction in the MIC values of levofloxacin was observed after the addition of CCCP (for ten isolates), PAβN (for seven isolates), and reserpine (for only one isolate). Moreover, three of the above-mentioned isolates (no. 44/2011, 47/2011, and 52/2012) showed a 4-fold reduction in the MIC values of levofloxacin in the presence of both CCCP and PAβN. For one isolate (no. 20/2011), a 4-fold increase in susceptibility to levofloxacin was observed in the presence of all three EPIs. The addition of CCCP to MH II medium reduced the MIC values of gentamicin (4-fold to at least a 32-fold) in 16 out of 94 isolates. Two of these 16 isolates (no. 15/2010 and 35/2011) also showed a 4-fold reduction in gentamicin MIC values in the presence of a second inhibitor, PAβN. Moreover, only one isolate (no. 42/2011) demonstrated the 4-fold reduction in the MIC values of gentamicin after the addition of reserpine.

The further investigations were performed on the 27 out of 94 isolates, for which at least a 4-fold reduction in the MIC values of levofloxacin or gentamicin was observed in the presence of any inhibitor (Table 2). For them, the effect of CCCP on the MIC values of following agents, trimethoprim/sulfamethoxazole, polymyxin B, chloramphenicol, erythromycin and tigecycline, was determined. The results were presented in Table S2 in Supplementary Materials, and in Table 2. In the presence of CCCP, a significant reduction in the MIC values of polymyxin B, from 5-fold to 16-fold, was observed for 4 out of 27 isolates. Additionally, one of these four isolates (no. 31/2011) also showed a 4-fold reduction in the MIC values of chloramphenicol. Besides, only one out of the group of 27 isolates (no. 41/2011) showed a significant reduction (6-fold) in the MIC values of trimethoprim/sulfamethoxazole after addition of CCCP. No changes in the susceptibility of the tested isolates to erythromycin or to tigecycline were obtained after adding CCCP to the test medium.

### 3.2. Expression of the Efflux Pump Genes

Six *S. maltophilia* isolates were chosen to analyze the expression level of genes encoding the following efflux systems: SmeDEF, SmeABC, SmeVWX, SmeGH, and SmeIJK. The selected isolates had a different susceptibility level to levofloxacin and also presented a significant reduction in the MIC values of: (I) only levofloxacin in the presence of all three investigated EPIs (no. 20/2011), (II) levofloxacin, and one other antimicrobial in the presence of one or two EPIs (no. 15/2010, 41/2011, and 52/2012), and (III) levofloxacin and two other antimicrobials in the presence of one or two EPIs (no. 31/2011 and 35/2011, respectively). The presence of efflux pump genes in the isolates selected for qPCR were included in Table S3 in Supplementary Materials.

Expression levels of *smeD, smeA, smeV, smeG*, and *smeI* genes relative to the susceptibility to levofloxacin in the presence or absence of EPIs are presented in Table 3. At least a 3-fold increase in efflux pump gene expression of tested isolates in comparison to levofloxacin-susceptible *S. maltophilia* 67/2013 was assessed as significant [35]. Only two out of five tested genes, *smeD* and *smeV*, were overexpressed in the selected isolates. The highest expression level of *smeV,* (60.81) was observed for *S. maltophilia* 41/2011. This isolate presented the highest MIC values of levofloxacin (MIC 16 mg/L). The highest expression level of *smeD* gene (21.40) was obtained in the case of *S. maltophilia* 15/2010, which was susceptible to levofloxacin. The 4-fold reduction in the MIC values of levofloxacin was observed in the presence of one EPI, CCCP for *S. maltophilia* 41/2011, and PAβN for *S. maltophilia* 15/2010.

### 3.3. Analysis of Efflux Systems Regulatory Genes

**Figure 1:**
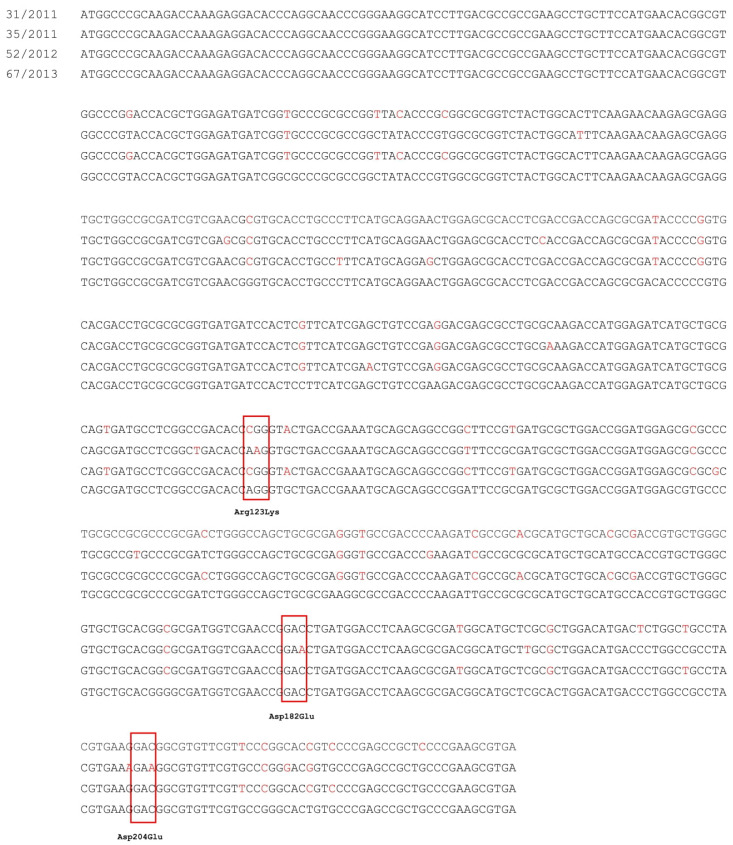
The point mutations of *smeT* gene in studied isolates in comparison to reference *S. maltophilia* 67/2013. Nucleotide mutations that led to amino acids changes are shown in the red boxes.

Analysis was performed for selected isolates, which presented the overexpression of *smeD* and *smeV* genes in comparison to levofloxacin-susceptible *S. maltophilia* 67/2013. Molecular detection by PCR revealed the presence of the *smeT* regulatory gene of the SmeDEF system in three studied isolates. The nucleotide sequences of *smeT* genes of isolates no. 31/2011, 35/2011, and 52/2012, in comparison to DNA of the reference isolate *S. maltophilia* 67/2013, are shown in Figure 1. Sequencing analysis revealed in all isolates from 27 to 35 nucleotide mutations in *smeT* in comparison to the reference isolate. However, the nucleotide mutations only led to the amino acid changes in one isolate, no. 35/2011 (AGG → AAG [Arg123Lys], GAC → GAA [Asp182Glu], and GAC → GAA [Asp204Glu]; Figure 1). The identical nucleotide sequences of *smeT* gene, as presented in isolates no. 35/2011 and 67/2013, were previously deposited in the GenBank NCBI database (accession no. CP067993.1, CP040440.1, and CP060026.1, respectively).

**Figure 2:**
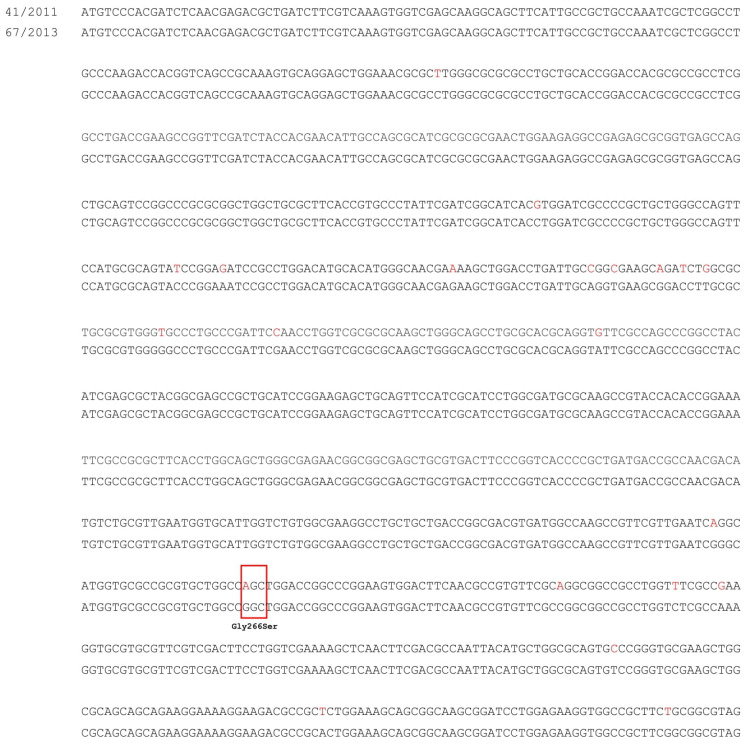
The point mutations of *smeRv* gene in studied *S. maltophilia* 41/2011 in comparison to reference *S. maltophilia* 67/2013. The nucleotide mutations that led to amino acids changes are shown in the red boxes.

The amplification of the *smeRv* regulatory gene of the SmeVWX system failed. Thus, the whole genome sequencing of *S. maltophilia* 41/2011 presented overexpression of *smeV* gene and the reference strain of *S. maltophilia* 67/2013 was performed. Sequencing analysis of the *smeRv* gene revealed 21 nucleotide mutations in the DNA of isolate no. 41/2011 in comparison to the reference strain, but only one of these mutations resulted in an amino acid change GGC → AGC (Gly266Ser). The nucleotide sequences of the *smeRv* gene of isolate no. 41/2011, in comparison to DNA of *S. maltophilia* 67/2013, are shown in Figure 2. The identical nucleotide sequence of *smeRv* as in reference strain 67/2013, is presented in the GenBank database (accession No. CP049956.1, CP040440.1, CP022053.2, CP060024.1, CP060026.1, AP021908.1, AM743169.1). The *smeRv* nucleotide sequence from 41/2011 strain has not been previously described.

## 4. Discussion

Levofloxacin, along with trimethoprim/sulfamethoxazole, is the main drug used in the treatment of *S. maltophilia* infection [9,10,11]. However, an increase in the number of resistant strains or strains with reduced susceptibility to this fluoroquinolone has been observed worldwide in the last two decades [9,12,13]. When considering the molecular basis of *S. maltophilia* clinical strains resistant to levofloxacin, the overproduction of RND efflux systems should be taken into account [15,18,26,27,35]. In this study, the contribution of efflux systems to levofloxacin resistance in 94 *S. maltophilia* clinical strains isolated in Warsaw was determined using phenotypic and molecular methods. Previously, the presence of genes encoding various ten efflux pumps from both RND and ABC families was shown in the majority of the 94 studied isolates [14]. To assess the role of efflux systems in antimicrobial resistance of *S. maltophilia*, three efflux pump inhibitors were used: CCCP, reserpine, and PAβN [42,43,44,45,46]. Among the 13 isolates for which a significant reduction in the MIC value of levofloxacin was demonstrated in the presence of EPIs, the majority of the isolates gave a positive result after the addition of CCCP. In the case of three isolates, the increase in susceptibility to this fluoroquinolone was obtained only after the addition of PAβN. It is known that CCCP inhibits the activity of all proton pumps in Gram-negative rods, including *S. maltophilia*. CCCP, as one of the protonophores, reduces ATP production and increases membrane permeability in bacteria by interfering with the transmembrane electrochemical gradient and proton motive force [47]. Unlike CCCP, other inhibitors used, such as PAβN and reserpine, have a narrower spectrum of activity. PAβN is an inhibitor of RND efflux pumps [43,45]. Reserpine is an indole alkaloid due to low specificity and affinity of reserpine-membrane glycoprotein binding. A high dose is required to inhibit some efflux pumps [44]. It should be emphasized that the activity of MDR efflux systems could be decreased by EPIs, but the relationship between the antibiotic MIC reduction in the presence of EPIs and efflux system overexpression is not always provided [42,43,44,45]. In addition, it has recently been shown that the simultaneous overexpression of the two efflux systems does not necessarily increase the resistance level to levofloxacin but results in the resistance to the extended spectrum of substrates of these systems [35]. Taking into account the above statements, the widespread occurrence of MDR efflux pumps in the tested isolates, up to 100%, and the possibility of overproduction of more than one efflux system in one isolate, we investigated the effect of CCCP, as a universal EPI, on the susceptibility of *S. maltophilia* to a wide spectrum of antibiotics. Only 5 out of 13 isolates (with the 4-fold reduction in levofloxacin MIC) showed an increased susceptibility to both levofloxacin and other antibiotics such as gentamicin, polymyxin B, or chloramphenicol in the presence of EPIs. On the other hand, for the majority of the isolates showing a significant reduction in the gentamicin MIC values in the presence of CCCP, such decreases were not obtained by testing other antibiotics, including levofloxacin.

The investigation of the role of MDR efflux pumps in resistance to antimicrobial agents is usually conducted by analyzing the expression levels of the efflux pump genes and studying regulatory genes [18,30,36]. In our study, five out of six investigated isolates overexpressed the *smeDEF* efflux system operon, but there was no correlation between the overexpression level, the resistance to levofloxacin, and the reduction of the MIC value of levofloxacin in the presence of EPIs. CCCP was the most effective EPI, which significantly reduced the levofloxacin MIC in each isolate with the overexpression of the *smeD* gene. It has been described that overexpression of the *smeDEF* operon is associated with mutations in its local regulatory gene, *smeT* [30,48]. The nucleotide mutation, which led to the amino acid change Arg123Lys, present in isolate no. 35/2011 in comparison to 67/2013, was previously described by Sanchez in urinary and sputum *S. maltophilia* isolates (C357 and E923, respectively) with an overproducing SmeDEF efflux system [48]. The second change in the amino acid sequence of SmeT found in isolate 35/2011 compared to isolate 67/2013 was Asp204Glu, while a similar change, Ala204Glu, was previously described in the *smeDEF*-overexpressed clinical isolate no. C357 [48]. Thus, it appears that the presence of Lys at position 123 and Glu at position 204 of the regulator protein contributes to the overexpression of the *smeDEF* operon. The third revealed amino acid change in SmeT of isolate no. 35/2011, in comparison to isolate no. 67/2013, was Asp182Glu. However, this change is not significant for the susceptibility profile of the strain. The presence of both Asp and Glu at position 182, previously observed in various isolates, was not associated with overexpression of the *smeDEF* operon [48]. An identical *smeT* nucleotide sequence, as present in 35/2011, was deposited in the GenBank NCBI database (accession no. CP067993.1). On the other hand, the same *smeT* gene sequence was demonstrated for the reference levofloxacin-sensitive isolate no. 67/2013 as well as for the levofloxacin-resistant *smeD*-overexpression clinical isolates. Therefore, the expression of the pump efflux operons in *S. maltophilia* is controlled not only by local regulators but also by global regulators. In other non-fermentative Gram-negative bacterium such as *Psudomonas aeruginosa*, the global regulator, SoxR, which affects the expression of genes encoding Mex efflux systems, has been described [49]. Moreover, in *S. maltophilia* strains, the presence of two-component regulatory systems and their influence on the expression of efflux pump operons was demonstrated. SmeSR involved in *smeABC* expression and the second TCS, SmeSyRy, regulated the *smeYZ* operon [26]. Recently, Wu et al. proved that inactivation of *smeRySy* not only decreased the expression of *smeYZ* but also increased the expression of the *smeDEF* operon [50]. Additionally, the obtained results of the expression levels of the *smeDEF* operons did not correlate with the susceptibility of the tested *S. maltophilia* isolates to levofloxacin. The levofloxacin-susceptible isolate no. 15/2010 showed the highest level of the *smeD* gene expression. On the other hand, the levofloxacin-resistant isolate no. 31/2011 exhibited the lowest level of this gene expression. Our results seem to confirm the observations of Wu et al. [35]. They indicated that simply assessing the overexpression of *smeDEF* operon by RT-qPCR did not significantly contribute to fluoroquinolone’s resistance. Moreover, for several investigated strains, deletion of *smeDEF* operons had no impact on their susceptibility. 

The second best-known efflux pump that contributes to the acquired fluoroquinolones-resistance of *S. maltophilia* strains is SmeVWX [18,36]. The overexpression of the *smeV* gene has been demonstrated by us in one isolate, no. 41/2011. Furthermore, this isolate was the most resistant to levofloxacin from the studied collection, and the 4-fold reduction in levofloxacin MIC in the presence of PAβN was shown. Recently, it was described that overexpression of the *smeVWX* operon is associated with mutations in the *smeRv* gene encoding the local regulator [27,30,36]. The change of SmeRv amino acid sequence in position 266, Gly266Ser, present in isolate no. 41/2011, has been previously described in several clinical strains isolated in Spain and in mutants obtained in vitro with the overproduction of the SmeVWX efflux system [27,30,36]. Moreover, the following changes in the amino acid sequence of the SmeRv regulator in *smeVWX*-overexpressing strains have been shown so far: Gly46Asp, Thr222Pro, Glu256Asp, Ala265Thr, Gly266Asp, Gly266Cys, and Cys310Phe. [27,30,36]. It should be emphasized that mutations leading to an amino acid change at position 266 of the regulatory protein were the most often detected mutation. They are extremely important for *smeVWX* overexpression, which contributes to drug resistance of such a strain. The strain PUC101, isolated in Spain (GenBank accession No. WP_180835982.1) with the same SmeRv amino acid sequence as Polish isolate no. 41/2011, was characterized by resistance to levofloxacin, colistin, ceftazidime, and ticarcillin-clavulanate, but there was no information concerning expression of the efflux system operon in PUC101 [51]. Besides that, only isolate no. 41/2011 showed a significant reduction in trimethoprim/sulfamethoxazole MIC in the presence of CCCP. This indicated that SmeVWX may also play an important role in resistance to trimethoprim/sulfamethoxazole, which is a known substrate of this efflux system.

As previously demonstrated, MDR efflux pumps are commonly found in clinical isolates [14]. Importantly, levofloxacin is a known substrate for 5 out of the 8 RND efflux systems. It is possible that the use of fluoroquinolones in monotherapy may lead to mutations in local and global regulatory genes. As a consequence, there are amino acid changes in the regulatory proteins, which causes the overexpression of the efflux system operons and the resistance of the strains.

## 5. Conclusions

Our data indicate that the SmeVWX efflux system plays a significant role in the resistance of clinical isolates to levofloxacin. Until now, mutations resulting in amino acid changes in the SmeRv regulatory protein have been reported in strains isolated in Spain. Analysis of the *smeRv* gene of the Polish isolate showed many point mutations and only one of them caused a significant change in the amino acid sequence, i.e., Gly266Ser. It should be emphasized that mutations leading to an amino acid change at position 266 of the regulatory protein were extremely important for the high level of *smeVWX* overexpression, which contributes to drug resistance of such a strain, especially in resistance to levofloxacin. However, our studies did not show a direct correlation between the level of susceptibility/resistance to levofloxacin of clinical isolates, the relative level of the *smeD* gene expression, and the sequence of the SmeT protein that regulates the activity of the *smeDEF* efflux system operon. Our data indicate that overexpression of SmeDEF did not play a significant role in levofloxacin resistance. It is likely that this system could be responsible for resistance to other antimicrobials.

## Figures and Tables

**Table 1:** Primers used for the analysis of MDR efflux pump gene expression by qPCR and for amplification of the efflux system regulatory genes.

Efflux System	Target Gene	Primer	Sequence (5’-3’)	Product Length (bp)	References	Purpose
SmeABC	*smeA*	A-F	AAGGCCATCGATGGCAAGGC	146	Zając et al. [14]	qPCR
		A-R	TCCGGGTTCGGAATGACCG			
SmeDEF	*smeD*	RT-D-F	CGGTCAGCATCCTGATGGA	73	Garcia-Leon et al. [28]	qPCR
		RT-D-R	ACGCTGACTTCGGAGAACTC			
SmeIJK	*smeI*	I-F	TTCCGCGAAGGCCAGGAAGT	107	Zając et al. [14]	qPCR
		I-R	TCGTTCTGGCGCTTGGCTG			
SmeVWX	*smeV*	V-F	ATGGCACGCAAGGGCGAG	118	This study	qPCR
		V-R	CCTGGTTGTCGAGGAAGTCG			
SmeGH	*smeG*	G-F	AAGAACGTGAAGACCGATGGC	107	Garcia-Leon et al. [28] modified	qPCR
		G-R	CCTTCCTTGACCTTCTGCAC		Garcia-Leon et al. [28]	
Not applicable	*gyrA*	gyrA-F	CAAGTCGGCG CGTATCGTC	82	This study	qPCR-internal control
		gyrA-R	GCGCACCAGC GTGTCGTA			
Not applicable	*rpoD*	rpoD-F	GCCGTACTGCTGGAGCAT	67	Bernardini et al. [41] modified	qPCR-internal control
		rpoD-R	GGTGCACATGATCGAAACGA		Bernardini et al. [41]	
Not applicable	*16S rDNA*	16S-F	GACCTTGCGCGATTGAATG	75	Zhao et al. [13]	qPCR-internal control
		16S-R	CGGATCGTCGCCTTGGT			
Regulatory gene of SmeDEF	*smeT*	smeT-F1	CCAGGATCACGGGGCTGTC	814	This study	PCR
		smeT-R1	TGCCACGCACACGACGGGAA			
		smeT-F2	ATGGCCCGCAAGACCAAAGAG	660		DNA sequencing
		smeT-R2	TCACGCTTCGGGCAGCGG			
Regulatory gene of SmeVWX	*smeRv*	smeRv-F1	CCCCGACGTCCAGGATCC	1121	Gracia-Leon et al. [27]	PCR
		smeRv-R1	GCTCGACTCTACAGAAGC			

F, the forward primer; R, the reverse primer.

**Table 2:** Effect of EPIs on the drug susceptibility of *S. maltophilia* clinical isolates (*n* = 27).

No	Isolate	MIC (mg/L)									
		LVX	LVX + CCCP	LVX + RES	LVX+ PAβN	PMB	PMB + CCCP	GEN	GEN + CCCP	GEN + RES	GEN+ PAβN
1	3/2010	4	4	4	4	1	1	>256	**128**	256	>256
2	8/2010	4	**1**	2	2	3	1	>256	>256	>256	>256
3	9/2010	16	8	8	**4**	2	1	>256	>256	>256	>256
4	10/2010	4	**1**	2	2	3	1	>256	>256	>256	>256
5	12/2010	2	2	1	2	1	0.5	256	**32**	256	256
6	15/2010 *	2	**0.5**	1	2	1	0.75	>256	**16**	>256	**128**
7	16/2010	2	2	1	2	1	0.5	256	**32**	256	256
8	17/2010	2	2	1	2	1	0.5	256	**32**	256	256
9	20/2011 *	4	**1**	**1**	**1**	1	1	128	64	128	128
10	22/2011	1	0.5	0.5	0.5	16	**3**	>256	**128**	256	256
11	24/2011	2	2	1	2	1	0.5	256	**32**	256	256
12	26/2011	2	2	1	2	1	0.5	256	**32**	256	256
13	31/2011 *	8	**2**	4	4	16	**1**	256	256	256	256
14	32/2011	1	0.5	0.5	**0.25**	1	0.75	>256	128	>256	>256
15	33/2011	4	**1**	4	4	0.75	0.5	>256	**64**	256	>256
16	34/2011	2	2	1	2	1	0.5	256	**32**	256	256
17	35/2011 *	2	**0.5**	1	1	6	**1**	>256	**128**	256	**128**
18	41/2011 *	16	8	8	**4**	1.5	0.75	>256	>256	>256	>256
19	42/2011	4	2	4	4	0.5	0.38	256	256	**64**	256
20	44/2011	8	**2**	4	**2**	0.5	0.5	256	256	256	256
21	47/2011	8	**2**	4	**2**	0.5	0.5	256	256	256	256
22	52/2012 *	8	**2**	4	**2**	3	**0.5**	>256	>256	>256	>256
23	56/2012	2	2	1	2	1	0.5	256	**32**	256	256
24	59/2012	2	2	1	2	1	0.5	256	**32**	256	256
25	61/2012	2	2	1	2	1	0.5	256	**32**	256	256
26	92/2013	2	2	2	2	1	0.38	256	**64**	256	256
27	95/2013	2	2	2	2	2	1	>256	**64**	>256	256

MIC, minimal inhibitory concentration; LVX, levofloxacin; PMB, polimyxin B; GEN, gentamicin; CCCP, cyanide 3-chlorophenylhydrazone; RES, reserpine; PAβN, phenylalanine-arginine β-naphthylamide. At least a 4-fold reduction in the MIC of antibiotic in the presence of EPI, when compared with the MIC values of antibiotic without EPI, is indicated in boldface. * Isolates selected for qPCR analysis of efflux pump gene expression.

**Table 3:** The expression level of efflux pump genes with regard to the susceptibility to levofloxacin in the presence or absence of EPIs.

Isolate	MIC of Levofloxacin [mg/L] (x-Fold Reduction in Levofloxacin MIC in the Presence of EPIs: CCCP, PAβN and Reserpine)/Susceptibility Interpretation	x-Fold Change ± SEM ^a^				
		*smeD*	*smeA*	*smeV*	*smeG*	*smeI*
15/2010	2 (4, 1, 2)/S	**21.40 ± 5.03**	0.09 ± 0.07	0.08 ± 0.0	0.01 ± 0.0	Not applicable ^b^
20/2011	4 (4, 4, 4)/I	**16.25 ± 3.02**	Not applicable ^b^	0.35 ± 0.04	0.01 ± 0.0	0.02 ± 0.0
31/2011	8 (4, 2, 2)/R	**3.47 ± 0.78**	0.03 ± 0.03	0.17 ± 0.04	2.53 ± 0.46	0.01 ± 0.0
35/2011	2 (4, 2, 2)/S	**10.75 ± 2.88**	Not applicable ^b^	0.0 ± 0.0	2.00 ± 0.14	Not applicable ^b^
41/2011	16 (2, 4, 2)/R	0.80 ± 0.11	0.78 ± 0.26	**60.81 ± 10.27**	1.55 ± 0.16	0.27 ± 0.03
52/2012	8 (4, 4, 2)/R	**15.31 ± 1.37**	2.09 ± 0.52	0.45 ± 0.07	2.79 ± 0.50	0.01 ± 0.0

SEM, standard error of mean value; MIC, the minimal inhibitory concentration; S-susceptible; R, resistant; I, intermediate. At least a 3-fold increase in a gene expression of the tested isolate, in comparison to levofloxacin-susceptible *S. maltophilia* 67/2013, is indicated in boldface. ^a^ Expression level of efflux pump genes are x-fold change of each strain versus the levofloxacin-susceptible *S. maltophilia* 67/2013. Data are the means from three independent experiments. ^b^ An efflux pump gene was not present in the studied isolate (Table S3 in Supplementary Materials).

## Supplementary Materials

The following are available online at https://www.mdpi.com/article/10.3390/biology11071044/s1, Table S1: Effect of EPIs on the susceptibility of *S. maltophilia* clinical isolates (*n* = 94) to levofloxacin and gentamycin, Table S2: Effect of CCCP on the drug susceptibility of *S. maltophilia* clinical isolates (*n* = 27), Table S3: Presence of efflux pump genes in *S. maltophilia* isolates selected for qPCR.
