# Supplementary material for: The Contribution of Efflux Systems to Levofloxacin Resistance in Stenotrophomonas maltophilia Clinical Strains Isolated in Warsaw, Poland
Source: Biology (Basel). 2022 Jul 12;11(7):1044. doi: 10.3390/biology11071044 (PMC9311822; doi:10.3390/biology11071044)
Supplement: Supplementary file 1 [file biology-11-01044-s001.zip › biology-1788060-supplementary.pdf]

Supplementary Material

The Contribution of Efflux Systems to Levofloxacin Resistance in *Stenotrophomonas maltophilia* Clinical Strains Isolated in Warsaw, Poland

Table S1. Effect of EPIs on the susceptibility of *S. maltophilia* clinical isolates (n = 94) to levofloxacin and gentamycin.

Isolates	MIC (mg/L)							
	LVX	LVX+CCCP	LVX+RES	LVX+PAβN	GEN	GEN+CCCP	GEN+RES	GEN+PAβN
1/2010	2	2	2	2	>256	>256	>256	>256
2/2010	4	2	4	4	128	64	64	128
3/2010	4	4	4	4	>256	**128**	256	>256
4/2010	4	4	4	4	256	256	256	256
5/2010	4	2	2	2	>256	>256	>256	>256
6/2010	4	4	4	4	>256	>256	>256	>256
7/2010	2	1	2	2	>256	>256	>256	256
8/2010	4	**1**	2	2	>256	>256	>256	256
9/2010	16	8	8	**4**	>256	>256	>256	>256
10/2010	4	**1**	2	2	>256	>256	>256	>256
11/2010	4	4	4	4	>256	>256	>256	>256
12/2010	2	2	1	2	256	**32**	256	256
13/2010	2	1	2	2	32	32	32	32
14/2010	4	4	2	2	>256	256	>256	>256
15/2010	2	**0,5**	1	2	>256	**16**	>256	**128**
16/2010	2	2	1	2	256	**32**	256	256
17/2010	2	2	1	2	256	**32**	256	256
18/2010	4	4	4	4	>256	>256	>256	>256

19/2010	4	2	4	2	>256	>256	>256	>256
20/2011	4	**1**	**1**	**1**	128	64	128	128
21/2011	2	2	2	2	128	128	128	128
22/2011	1	0,5	0,5	0,5	>256	**128**	256	256
23/2011	4	4	4	4	>256	>256	>256	>256
24/2011	2	2	1	2	256	**32**	256	256
25/2011	2	1	2	2	256	128	128	256
26/2011	2	2	1	2	256	**32**	256	256
27/2011	2	1	2	2	256	128	128	256
28/2011	4	2	2	4	>256	>256	>256	>256
29/2011	4	4	4	4	128	128	128	128
30/2011	16	16	16	16	16	16	16	16
31/2011	8	**2**	4	4	256	256	256	256
32/2011	1	0,5	0,5	**0,25**	>256	128	>256	>256
33/2011	4	**1**	4	4	>256	**64**	256	>256
34/2011	2	2	1	2	256	**32**	256	256
35/2011	2	**0,5**	1	1	>256	**128**	256	**128**
36/2011	4	2	2	2	256	128	256	256
37/2011	4	4	4	4	>256	>256	>256	>256
38/2011	4	4	4	4	>256	>256	>256	>256
39/2011	2	2	2	2	>256	>256	>256	>256
41/2011	16	8	8	**4**	>256	>256	>256	>256
42/2011	4	2	4	4	256	256	**64**	256
43/2011	2	1	2	2	>256	>256	>256	>256

44/2011	8	**2**	4	**2**	256	256	256	256
45/2011	2	2	2	2	>256	256	>256	>256
46/2011	2	2	2	2	>256	>256	>256	>256
47/2011	8	**2**	4	**2**	256	256	256	256
48/2011	2	1	2	2	>256	>256	>256	>256
49/2011	4	2	4	4	>256	256	>256	256
50/2012	2	1	2	2	256	256	256	256
51/2012	2	2	2	2	>256	>256	>256	>256
52/2012	8	**2**	4	**2**	>256	>256	>256	>256
53/2012	2	2	2	2	>256	256	>256	>256
54/2012	4	4	4	4	>256	>256	>256	>256
55/2012	2	2	2	2	>256	>256	>256	>256
56/2012	2	2	1	2	256	**32**	256	256
57/2012	2	2	2	2	>256	>256	>256	>256
58/2012	4	4	4	4	256	256	256	256
59/2012	2	2	1	2	256	**32**	256	256
60/2012	2	2	2	2	>256	>256	>256	>256
61/2012	2	2	1	2	256	**32**	256	256
62/2012	2	1	2	2	256	128	128	256
63/2012	2	1	2	2	256	128	128	256
64/2012	4	4	4	4	>256	>256	>256	>256
65/2012	4	4	4	4	>256	>256	>256	>256
66/2013	4	4	4	4	>256	>256	256	256
67/2013	1	1	1	1	64	64	64	64

68/2013	4	4	2	2	>256	>256	>256	256
69/2013	2	1	2	2	256	128	128	256
70/2013	1	1	1	1	>256	>256	>256	>256
71/2013	4	2	2	4	>256	>256	>256	>256
72/2013	2	2	2	2	>256	256	>256	>256
73/2013	4	4	4	4	>256	>256	>256	>256
74/2013	1	0,5	1	1	>256	>256	>256	>256
75/2013	2	1	1	2	128	128	128	64
76/2013	2	2	1	2	>256	>256	>256	>256
77a/2013	4	2	4	4	256	256	256	256
77b/2013	4	4	4	4	>256	>256	256	>256
78a/2013	2	1	2	2	128	128	128	128
78b/2013	2	2	2	2	256	256	256	256
79/2013	2	2	2	1	128	64	128	128
80/2013	4	4	2	2	>256	>256	>256	>256
81/2013	2	2	2	2	>256	>256	>256	>256
82/2013	2	2	2	2	8	8	8	4
83/2013	4	4	4	4	>256	>256	>256	>256
84/2013	4	4	4	4	128	128	128	128
85/2011	4	4	4	4	256	256	256	256
88/2011	2	1	2	2	256	128	128	256
89/2011	2	2	2	2	>256	>256	>256	>256
90/2011	2	2	2	2	256	128	256	256
91/2013	4	4	4	4	>256	>256	>256	>256

92/2013	2	2	2	2	256	**64**	256	256
93/2013	4	2	2	4	>256	>256	>256	>256
94/2013	2	2	2	2	128	128	128	128
95/2013	2	2	2	2	>256	**64**	>256	256

MIC, minimal inhibitory concentration; LVX, levofloxacin; PMB, polimyxin B; GEN, gentamicin; CCCP, cyanide 3-chlorophenylhydrazone; RES, reserpine; PAβN, phenylalanine-arginine β-naphthylamide. At least a 4-fold reduction in the MIC of antibiotic in the presence of EPI, when compared with the MIC values of antibiotic without EPI, is indicated in boldface.

Table S2. Effect of CCCP on the drug susceptibility of *S. maltophilia* clinical isolates (n = 27).

No	Isolates	Clinical material	MIC (mg/L)									
			TMP/SMX	TMP/SMX+CCCP	PMB	PMB +CCCP	CHL	CHL +CCCP	ERY	ERY +CCCP	TIG	TIG +CCCP
1	3/2010	oral cavity swab	0,094	0,064	1	1	32	32	>256	>256	1,5	1,5
2	8/2010	stoma swab	0,125	0,125	3	1	24	15	>256	>256	0,75	0,75
3	9/2010	anus swab	0,094	0,064	2	1	32	16	>256	>256	1,0	0,75
4	10/2010	peritoneal fluid	0,094	0,047	3	1	24	12	>256	>256	1,5	1,0
5	12/2010	blood	0,125	0,125	1	0,5	48	32	>256	>256	0,75	0,75
6	15/2010*	blood	0,25	0,125	1	0,75	32	32	>256	>256	0,75	0,75
7	16/2010	blood	0,125	0,125	1	0,5	48	32	>256	>256	0,75	0,75
8	17/2010	blood	0,125	0,125	1	0,5	48	32	>256	>256	0,75	0,75
9	20/2011*	anus swab	0,094	0,094	1	1	12	12	>256	>256	0,75	0,75
10	22/2011	bronchial secretion	0,094	0,094	16	**3**	12	8	64	48	0,5	0,5
11	24/2011	blood	0,125	0,125	1	0,5	48	32	>256	>256	0,75	0,75
12	26/2011	blood	0,125	0,125	1	0,5	48	32	>256	>256	0,75	0,75
13	31/2011*	urine sample	0,125	0,094	16	**1**	128	**32**	96	96	1,5	1
14	32/2011	urine sample	0,064	0,047	1	0,75	8	6	16	8	0,38	0,19
15	33/2011	blood	0,19	0,19	0,75	0,5	12	12	>256	>256	0,75	0,38
16	34/2011	blood	0,125	0,125	1	0,5	48	32	>256	>256	0,75	0,75
17	35/2011*	urine sample	0,125	0,125	6	**1**	32	32	>256	>256	0,5	0,38
18	41/2011*	urine	0,75	**0,125**	1,5	0,75	>256	>256	48	48	0,38	0,38

		sample										
19	42/2011	gastrostomy swab	0,047	0,047	0,5	0,38	12	12	>256	>256	0,75	0,5
20	44/2011	anus swab	0,19	0,125	0,5	0,5	32	32	>256	>256	1	0,75
21	47/2011	bronchial secretion	0,19	0,125	0,5	0,5	32	32	>256	>256	1	0,75
22	52/2012*	urine sample	0,19	0,19	3	**0,5**	16	16	>256	>256	0,75	0,5
23	56/2012	blood	0,125	0,125	1	0,5	48	32	>256	>256	0,75	0,75
24	59/2012	blood	0,125	0,125	1	0,5	48	32	>256	>256	0,75	0,75
25	61/2012	blood	0,125	0,125	1	0,5	48	32	>256	>256	0,75	0,75
26	92/2013	blood	0,38	0,38	1	0,38	16	16	>256	>256	0,5	0,38
27	95/2013	bronchial secretion	0,19	0,19	2	1	48	32	>256	>256	0,75	0,75

TMP/SMX, trimethoprim/sulfamethoxazole; PMB, polimyxin B; CHL, chloramphenicol; ERY, erythromycin; TIG, tigecyclin; CCCP, cyanide 3-chlorophenylhydrazone; RES, reserpine; PAβN, phenylalanine-arginine β-naphthylamide. At least a 4-fold reduction in the MIC of antibiotic in the presence of EPI, when compared with the MIC values of antibiotic without EPI, is indicated in boldface.
*isolates selected for qPCR.

Table S3. Presence of efflux pump genes in *S. maltophilia* isolates selected for qPCR [1].

**Strain**	*smeD*	*smeB*	*smeW*	*smeH*	*smeK*
15/2010	+	+	+	+	-
20/2011	+	-	+	+	+
31/2011	+	+	+	+	+
35/2011	+	-	+	+	-
41/2011	+	+	+	+	+
52/2012	+	+	+	+	+
67/2013	+	+	+	+	+

+, positive result of gene amplification; -, negative result of gene amplification.

References

1. Zając, O.M.; Tyski, S.; Laudy, A.E. Phenotypic and Molecular Characteristics of the MDR Efflux Pump Gene-Carrying Stenotrophomonas maltophilia Strains Isolated in Warsaw, Poland. *Biology* **2022**. *11*, 105. https://doi.org/10.3390/biology11010105
